# Impact on genetic differences among various chicken breeds on free amino acid contents of egg yolk and albumen

**DOI:** 10.1038/s41598-021-81660-3

**Published:** 2021-01-26

**Authors:** Tatsuhiko Goto, Saki Shimamoto, Masahiro Takaya, Shun Sato, Kanna Takahashi, Kenji Nishimura, Yasuko Morii, Kyoko Kunishige, Akira Ohtsuka, Daichi Ijiri

**Affiliations:** 1 Research Center for Global Agromedicine, Obihiro University of Agriculture and Veterinary Medicine, Obihiro, Hokkaido 080-8555 Japan; 2 Department of Life and Food Sciences, Obihiro University of Agriculture and Veterinary Medicine, Obihiro, Hokkaido 080-8555 Japan; 3 Department of Biochemical Science and Technology, Kagoshima University, Korimoto, Kagoshima 890-0065 Japan; 4 Graduate School of Science and Technology, Niigata University, Niigata, 950-2181 Japan; 5 Hokkaido Tokachi Area Regional Food Processing Technology Center, Tokachi Foundation, Obihiro, Hokkaido 080-2462 Japan; 6 Agricultural Research Department, Animal Research Center, Hokkaido Research Organization, Shintoku, Hokkaido 081-0038 Japan

**Keywords:** Agricultural genetics, Animal breeding, Genotype

## Abstract

Eggs play important roles as food resources and nutraceuticals, to alleviate malnutrition and to improve health status in the world. Since free amino acids contribute to the nutritional values and food tastes, we investigated a total of 81 eggs from five chicken breeds, which are Australorp, Nagoya (NGY), Rhode Island Red (RIR), Shamo (SHA), Ukokkei, and two F_1_ hybrids (NGYxRIR and SHAxRIR) to test impact on genetic differences in 10 egg traits, 20 yolk amino acid traits, and 18 albumen amino acid traits. One-way ANOVA revealed significant breed effects on 10 egg traits, 20 yolk amino acid traits, and 15 albumen amino acid traits. Moreover, a significant heterosis effect on yolk aspartic acid was identified. In addition, positive correlations were found broadly among traits within each trait category (egg traits, yolk amino acid traits, and albumen amino acid traits), whereas there were basically no or weak correlations among the trait categories. These results suggest that almost all traits can be dramatically modified by genetic factor, and there will be partially independent production systems of amino acids into yolk and albumen. Since there will be typical quantitative genetic architecture of egg contents, further genetic analyses will be needed.

## Introduction

Egg from hens is one of the most abundant livestock products in the world^1^. Eggs are well known as important sources of human nutrients, containing proteins, lipids, vitamins, minerals, and growth factors for the embryos^2^. Nearly 1000 yolk proteins and 150 albumen proteins have been identified, and these at least provide us essential amino acids^3^. Moreover, egg components have several biological activities, including antioxidant properties, antimicrobial activities, immunomodulatory, anticancer, and antihypertensive activities^2,3^. These important characteristics of eggs as food resources and nutraceuticals will enhance utility not only alleviating malnutrition and hunger situation in developing countries^4^ but also improving health status for children and aged people in developed countries.

Egg traits such as egg weight, eggshell thickness, yolk size, and albumen weight are directly related to the quality and quantity of egg products. Therefore, many researchers have been investigated for eggshell^5^, yolk^6^, and albumen^7^ at molecular level. Several factors affecting egg traits have been summarized^8^. Of them, genetic factors, especially many quantitative trait loci (QTLs), have been identified for regulating size, weight, and color of the egg^9–11^. Although there will be the similar complex genetic basis of molecular contents in eggs as well as size and weight of the egg^12^, little is known about the genetic factor. Until now, there are some researches to investigate the genetic factor of egg contents focusing on fatty acids and minerals^13–15^, free amino acids^16^, and metabolites^17^.

Recent poultry industry in the world has used specific breeds of chickens, which are White Leghorn, Rhode Island Red, and White Plymouth Rock for egg layers, and White Cornish and White Plymouth Rock for broilers, and all commercial breeding stocks utilize heterosis by crossing different strains^18^. Although their industrial chickens indicate remarkable productivity^19^, its genetic diversity is limited from poultry genetic resources including standardized breeds and village chickens in the world^20^. In Japan, Tsudzuki^21^ has introduced Japanese indigenous chicken breeds, which are consisting of approximately 50 breeds. In addition, hybrid vigour based on the crosses between Japanese fancy breeds (e.g., Oh-Shamo, Hinai-dori, and Nagoya) and American breeds (e.g., White Plymouth Rock and Rhode Island Red) has been used frequently to produce more delicious meat as Jidori brand^21,22^. In this study, five breeds (Australorp; AUS, Nagoya; NGY, Rhode Island Red; RIR, Shamo; SHA, and Ukokkei; UKO)^23^ and two F_1_ hybrids (NGYxRIR and SHAxRIR) of chickens were investigated. Especially, SHA, NGY, and RIR have been used to create three-way crossbred, Shintoku-Jidori chickens in Hokkaido Research Organization, Japan. Large genetic differences among various chicken breeds and heterosis will have a large potential to enhance egg contents at molecular level.

A potential for enhancing the nutritional values by using genetic and environmental factors have been reported in egg free amino acids^16,24^. Since free amino acids contribute to the taste of foodstuffs^25,26^, fundamental information of amino acids contents in eggs will lead to increase not only the nutritional values but also food tastes. Until now, there are few reports in phenotypic correlations between egg size-related traits and egg amino acid traits. Therefore, in this study, we aimed to reveal breed and heterosis effects on yolk and albumen amino acids contents and phenotypic correlations between general egg traits and egg amino acid traits using five breeds (AUS, NGY, RIR, SHA, and UKO) and two F_1_ hybrids (NGYxRIR and SHAxRIR) of chickens.

## Results

### General egg traits

To determine the effect of genetic difference, we measured 10 egg traits: egg weight (EW), length of the long axis of egg (LLE), length of the short axis of egg (LSE), eggshell weight (SW), yolk weight (YW), albumen weight (AW), eggshell thickness (ST), eggshell color lightness (SCL), eggshell color redness (SCR), and eggshell color yellowness (SCY) using AUS, NGY, RIR, SHA, UKO, NGYxRIR, and SHAxRIR chickens (Table 1 and Supplementary Figure S1). One-way analysis of variance (ANOVA) revealed significant breed effects on EW, LLE, and LSE (F_6,70_ = 32.3, 16.7, and 20.1, *p* = 2.20E−16, 7.26E−12, and 1.60E−13, respectively). NGYxRIR and SHA laid larger eggs, whereas UKO produced smaller eggs. There were significant breed effects on AW and YW (F_6,70_ = 20.2 and 14.6, *p* = 1.43E−13 and 9.59E−11, respectively). NGYxRIR and RIR were higher AW values, whereas SHA was highest YW value. UKO was lowest in AW and YW. Significant breed effects were found in SW and ST (F_6,70_ = 20.4 and 4.1, *p* = 1.14E−13 and 1.48E−03, respectively). NGYxRIR and UKO indicated highest and lowest values in SW and ST, respectively. One-way ANOVA discovered significant breed effects on SCL, SCR, and SCY (F_6,70_ = 27.4, 22.8, and 13.4, *p* = 2.20E−16, 9.79E−15, and 5.05E−10, respectively). RIR and SHAxRIR showed lower lightness and higher redness and yellowness in eggshell, while NGY and UKO indicated lighter and paler eggshell color.

**Table 1:** All egg traits and amino acid traits in five breeds and two F_1_ hybrids of chickens.

Trait	AUS	NGY	NGYxRIR	RIR	SHAxRIR	SHA	UKO	*p* value
EW (g)	54.4 ± 3.6	57.5 ± 3.8	59.6 ± 5.0	56.8 ± 3.7	58.6 ± 4.8	60.3 ± 2.8	41.8 ± 2.8	***
LLE (mm)	58.9 ± 2.8	58.2 ± 1.7	59.6 ± 2.8	57.5 ± 2.2	57.2 ± 2.3	59.4 ± 2.8	51.2 ± 1.8	***
LSE (mm)	42.3 ± 0.7	42.7 ± 1.0	43.4 ± 0.8	43.4 ± 1.6	43.3 ± 1.4	43.3 ± 1.1	39.3 ± 1.2	***
AW (g)	30.6 ± 2.9	30.4 ± 2.7	32.5 ± 3.7	32.5 ± 3.6	29.6 ± 3.0	29.9 ± 0.7	21.7 ± 1.6	***
YW (g)	17.2 ± 1.4	18.0 ± 1.6	19.7 ± 1.9	18.1 ± 1.5	19.5 ± 1.5	20.9 ± 1.5	15.4 ± 1.7	***
SW (g)	6.5 ± 0.7	6.2 ± 0.7	7.4 ± 1.0	6.6 ± 0.5	7.0 ± 0.5	7.0 ± 0.9	4.7 ± 0.5	***
ST (mm)	0.42 ± 0.0	0.40 ± 0.1	0.47 ± 0.1	0.41 ± 0.0	0.46 ± 0.0	0.43 ± 0.0	0.38 ± 0.0	**
SCL	74.7 ± 3.9	82.2 ± 3.8	67.9 ± 5.1	63.5 ± 3.1	67.7 ± 5.7	78.6 ± 6.5	81.1 ± 3.2	***
SCR	8.4 ± 2.1	5.3 ± 2.0	12.5 ± 3.8	15.5 ± 1.8	14.1 ± 3.4	7.0 ± 4.4	5.4 ± 2.6	***
SCY	18.1 ± 3.5	15.2 ± 3.8	19.6 ± 3.4	25.5 ± 1.5	25.7 ± 3.0	19.5 ± 6.4	16.6 ± 3.5	***
Y_Asp	31.9 ± 5.3	51.6 ± 7.0	36.2 ± 8.3	47.2 ± 9.2	57.5 ± 9.1	48.8 ± 7.4	43.2 ± 6.6	***
Y_Glu	196.9 ± 9.7	205.6 ± 10.4	175.2 ± 12.2	182.9 ± 18.5	216.0 ± 17.5	221.0 ± 15.8	195.6 ± 17.0	***
Y_Asn	38.8 ± 2.1	26.8 ± 2.6	30.0 ± 2.2	34.6 ± 4.1	35.9 ± 2.9	34.4 ± 2.0	35.0 ± 2.6	***
Y_Ser	64.8 ± 2.4	57.2 ± 2.8	54.9 ± 4.1	59.1 ± 5.1	60.5 ± 4.5	58.2 ± 4.6	60.6 ± 3.4	***
Y_Gln	65.4 ± 2.6	56.5 ± 4.8	58.2 ± 5.0	61.8 ± 4.8	63.9 ± 3.7	60.6 ± 2.3	58.1 ± 1.8	***
Y_Gly	23.4 ± 1.2	18.6 ± 1.1	19.2 ± 1.6	20.7 ± 2.1	21.9 ± 2.2	21.9 ± 1.9	21.9 ± 1.7	***
Y_His	24.2 ± 1.7	18.8 ± 1.8	17.4 ± 2.5	20.2 ± 2.4	23.4 ± 3.9	22.5 ± 3.2	22.6 ± 1.9	***
Y_Arg	95.4 ± 4.7	77.1 ± 5.9	76.8 ± 8.8	85.0 ± 10.2	91.2 ± 6.3	89.4 ± 6.4	90.7 ± 6.6	***
Y_Thr	57.2 ± 2.9	51.3 ± 3.5	50.3 ± 4.7	51.3 ± 3.1	55.7 ± 4.5	59.3 ± 4.4	67.6 ± 13.1	***
Y_Ala	42.2 ± 1.8	33.8 ± 2.0	36.5 ± 3.8	37.9 ± 4.1	39.0 ± 3.0	38.7 ± 3.4	39.1 ± 3.2	***
Y_Pro	41.6 ± 1.9	34.4 ± 2.2	35.2 ± 3.3	38.0 ± 4.0	38.7 ± 2.6	38.8 ± 1.9	42.1 ± 2.1	***
Y_GABA	1.1 ± 0.3	1.2 ± 0.5	1.0 ± 0.5	1.1 ± 0.5	1.2 ± 0.5	1.3 ± 0.4	1.6 ± 0.5	*
Y_Tyr	83.3 ± 3.7	73.4 ± 3.3	72.5 ± 6.9	77.5 ± 6.5	81.5 ± 5.7	78.5 ± 5.3	80.4 ± 4.8	***
Y_Val	70.5 ± 3.0	58.0 ± 4.6	56.0 ± 5.4	60.8 ± 4.4	65.8 ± 4.2	65.5 ± 5.4	68.2 ± 1.9	***
Y_Met	34.8 ± 2.1	25.4 ± 2.1	26.8 ± 2.7	29.0 ± 2.2	28.8 ± 2.1	30.7 ± 2.4	32.1 ± 2.3	***
Y_Cys	4.3 ± 0.7	3.7 ± 0.8	3.7 ± 0.6	3.1 ± 0.7	3.6 ± 0.8	3.4 ± 0.5	3.4 ± 0.8	*
Y_Ile	61.7 ± 3.9	50.2 ± 3.7	48.3 ± 5.4	51.8 ± 5.5	55.9 ± 3.7	55.1 ± 4.2	58.7 ± 3.2	***
Y_Leu	130.4 ± 4.8	104.5 ± 6.5	106.4 ± 10.7	115.4 ± 12.6	121.6 ± 8.0	121.0 ± 10.6	123.0 ± 5.6	***
Y_Phe	67.4 ± 3.7	52.0 ± 3.2	52.1 ± 5.4	57.4 ± 5.8	61.1 ± 3.9	60.8 ± 4.2	64.2 ± 3.7	***
Y_Lys	114.0 ± 5.5	93.8 ± 6.4	90.6 ± 9.6	96.4 ± 9.8	108.3 ± 10.0	107.5 ± 9.6	107.8 ± 7.9	***
A_Asp	23.3 ± 3.9	30.0 ± 9.0	29.6 ± 4.3	26.0 ± 2.7	31.6 ± 4.6	36.1 ± 5.1	35.5 ± 10.6	***
A_Glu	36.0 ± 6.1	49.7 ± 16.3	46.4 ± 5.7	37.7 ± 4.3	49.7 ± 7.3	57.9 ± 7.6	60.4 ± 18.1	***
A_Ser	22.9 ± 4.4	29.1 ± 7.3	27.8 ± 5.0	23.5 ± 3.0	27.5 ± 3.4	31.4 ± 5.7	36.0 ± 10.9	***
A_Gln	16.7 ± 5.2	17.2 ± 8.6	21.4 ± 6.9	18.2 ± 8.7	14.1 ± 6.5	16.8 ± 9.2	17.5 ± 10.2	ns
A_His	6.4 ± 4.8	6.1 ± 3.8	4.6 ± 2.7	6.3 ± 4.5	6.7 ± 3.7	7.3 ± 3.7	8.1 ± 4.1	ns
A_Gly	4.0 ± 1.6	4.1 ± 1.6	3.1 ± 1.5	3.6 ± 1.3	2.8 ± 1.3	2.9 ± 1.0	7.0 ± 3.5	***
A_Thr	19.0 ± 3.2	25.9 ± 4.4	22.5 ± 2.4	19.0 ± 2.5	22.9 ± 2.8	27.2 ± 2.6	37.0 ± 11.7	***
A_Arg	18.8 ± 3.0	21.5 ± 7.0	19.4 ± 3.2	19.7 ± 2.6	23.3 ± 3.4	26.1 ± 4.3	29.5 ± 8.3	***
A_Ala	22.1 ± 3.6	26.5 ± 9.6	26.9 ± 4.8	22.3 ± 4.3	27.4 ± 4.9	32.2 ± 4.1	34.7 ± 9.9	***
A_Tyr	12.7 ± 2.2	16.7 ± 5.1	15.7 ± 2.4	13.3 ± 1.4	16.3 ± 1.5	17.5 ± 2.5	19.6 ± 5.7	***
A_Val	21.4 ± 3.8	26.4 ± 3.7	23.2 ± 3.5	21.6 ± 2.7	26.7 ± 2.2	29.9 ± 3.9	32.6 ± 9.3	***
A_Met	13.5 ± 1.9	15.0 ± 3.2	13.8 ± 2.2	12.4 ± 2.0	15.4 ± 1.5	17.0 ± 2.5	19.1 ± 5.5	***
A_Trp	2.5 ± 0.4	2.9 ± 0.5	3.1 ± 0.5	2.6 ± 0.4	3.0 ± 0.4	3.4 ± 0.6	3.6 ± 1.1	***
A_Phe	12.0 ± 4.3	15.6 ± 2.4	13.5 ± 2.8	12.0 ± 1.7	15.5 ± 1.6	15.6 ± 2.0	19.2 ± 5.2	***
A_Ile	16.2 ± 4.3	17.9 ± 4.7	16.1 ± 2.9	14.7 ± 2.1	18.9 ± 2.3	20.2 ± 2.7	23.3 ± 7.1	***
A_Leu	34.0 ± 5.0	39.2 ± 10.9	38.1 ± 6.4	33.6 ± 4.2	42.1 ± 4.9	45.0 ± 5.0	49.1 ± 14.5	***
A_Lys	0.8 ± 0.4	0.8 ± 0.4	0.7 ± 0.3	0.6 ± 0.3	0.8 ± 0.4	0.8 ± 0.3	1.1 ± 0.4	ns
A_Pro	26.8 ± 4.8	32.3 ± 9.1	28.6 ± 8.5	25.9 ± 3.6	34.0 ± 4.1	37.7 ± 4.0	40.5 ± 11.9	***

Trait abbreviations were shown in methods. Amino acid concentrations were shown in µg/mL. *p* value from one-way ANOVA is shown as ***p < 0.001, **p < 0.01, *p < 0.05, and ^ns^*p* > 0.05. Mean ± SD. Results of Tukey’s HSD tests were shown in Figures S1,S2, and S3.

### Yolk amino acids traits

This study detected a total of 20 yolk free amino acids: aspartic acid (Y_Asp), glutamic acid (Y_Glu), asparagine (Y_Asn), serine (Y_Ser), glutamine (Y_Gln), glycine (Y_Gly), histidine (Y_His), arginine (Y_Arg), threonine (Y_Thr), alanine (Y_Ala), proline (Y_Pro), gamma-aminobutyric acid (Y_GABA), tyrosine (Y_Tyr), valine (Y_Val), methionine (Y_Met), cysteine (Y_Cys), isoleucine (Y_Ile), phenylalanine (Y_Phe), and lysine (Y_Lys). As shown in Table 1 and Supplementary Figure S2, one-way ANOVA revealed significant breed effects on all 20 yolk amino acid traits: Y_Asp (F_6,70_ = 15.5, *p* = 3.27E−11), Y_Glu (F_6,70_ = 12.7, *p* = 1.26E−09), Y_Asn (F_6,70_ = 24.5, *p* = 1.96E−15), Y_Ser (F_6,70_ = 8.3, *p* = 8.52E−07), Y_Gln (F_6,70_ = 9.2, *p* = 1.94E−07), Y_Gly (F_6,70_ = 11.6, *p* = 5.97E−09), Y_His (F_6,70_ = 10.3, *p* = 4.04E−08), Y_Arg (F_6,70_ = 12.2, *p* = 2.57E−09), Y_Thr (F_6,70_ = 10.6, *p* = 2.43E−08), Y_Ala (F_6,70_ = 8.8, *p* = 3.68E−07), Y_Pro (F_6,70_ = 14.0, *p* = 2.15E−10), Y_GABA (F_6,70_ = 2.3, *p* = 0.044), Y_Tyr (F_6,70_ = 6.8, *p* = 9.57E−06), Y_Val (F_6,70_ = 18.9, *p* = 6.09E−13), Y_Met (F_6,70_ = 24.2, *p* = 2.71E−15), Y_Cys (F_6,70_ = 2.9, *p* = 0.014), Y_Ile (F_6,70_ = 14.9, *p* = 7.11E−11), Y_Leu (F_6,70_ = 15.4, *p* = 3.60E−11), Y_Phe (F_6,70_ = 23.0, *p* = 8.41E−15), and Y_Lys (F_6,70_ = 12.2, *p* = 2.28E−09). AUS indicated higher yolk amino acid contents, whereas NGY and NGYxRIR showed lower contents in almost all yolk amino acid traits except for Y_Asp, Y_Glu, and Y_GABA.

### Albumen amino acids traits

This study detected a total of 18 albumen free amino acids: aspartic acid (A_Asp), glutamic acid (A_Glu), serine (A_Ser), glutamine (A_Gln), histidine (A_His), glycine (A_Gly), threonine (A_Thr), arginine (A_Arg), alanine (A_Ala), tyrosine (A_Tyr), valine (A_Val), methionine (A_Met), tryptophan (A_Trp), phenylalanine (A_Phe), isoleucine (A_Ile), leucine (A_Ieu), lysine (A_Lys), and proline (A_Pro) as shown in Table 1 and Supplementary Figure S3. Of 15 albumen amino acid traits, one-way ANOVA revealed significant breed effects on A_Asp (F_6,70_ = 5.9, *p* = 5.39E−05), A_Glu (F_6,70_ = 7.7, *p* = 2.45E−06), A_Ser (F_6,70_ = 5.8, *p* = 6.10E−05), A_Gly (F_6,70_ = 7.1, *p* = 6.36E−06), A_Thr (F_6,70_ = 15.7, *p* = 2.40E−11), A_Arg (F_6,70_ = 7.3, *p* = 4.52E−06), A_Ala (F_6,70_ = 5.8, *p* = 6.31E−05), A_Tyr (F_6,70_ = 5.8, *p* = 5.84E−05), A_Val (F_6,70_ = 8.8, *p* = 3.85E−07), A_Met (F_6,70_ = 6.3, *p* = 2.55E−05), A_Trp (F_6,70_ = 5.6, *p* = 8.63E−05), A_Phe (F_6,70_ = 7.3, *p* = 4.28E−06), A_Ile (F_6,70_ = 5.5, *p* = 1.12E−04), A_Leu (F_6,70_ = 5.1, *p* = 2.21E−04), and A_Pro (F_6,70_ = 6.3, *p* = 2.29E−05). Of them, SHA and UKO showed higher contents of albumen amino acids, while AUS and RIR indicated lower contents. On the other hand, there were no significant breed effects in the remaining three traits: A_Gln (F_6,70_ = 0.8, *p* = 0.538), A_His (F_6,70_ = 0.9, *p* = 0.505), and A_Lys (F_6,70_ = 2.1, *p* = 0.061).

### Phenotypic correlations

**Figure 1:**
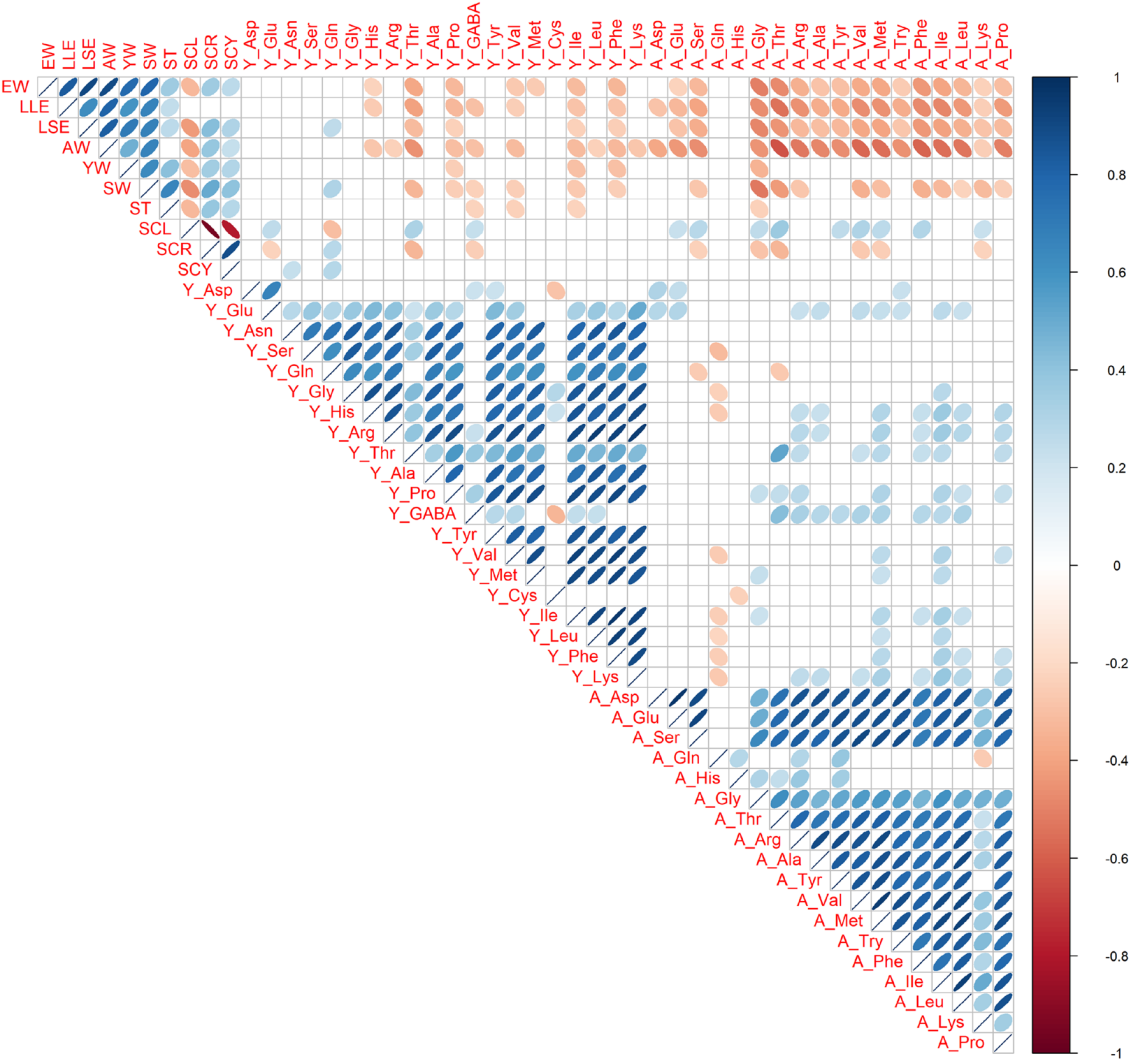
Phenotypic correlations among all 48 egg traits and free amino acid traits. Ten egg traits, 20 yolk amino acids traits, and 18 albumen amino acids traits from a total of 81 hens (AUS, NGY, RIR, SHA, UKO, NGYxRIR, and SHAxRIR) were used. Trait abbreviations are shown in Methods. Pearson’s correlations are expressed by ellipses. Blue and red ellipses indicate positive and negative correlations in each pair (*p* < 0.05). Blank cells show not significance.

Pair-wise Pearson’s correlation coefficients among 48 traits were estimated in Fig. 1. In 10 egg traits, there were moderate to strong positive correlations among sizes and weights (EW, LLE, LSE, AW, YW, and SW). Among eggshell color traits, strong positive correlation was estimated between SCR and SCY, but negative correlations were estimated between lightness (SCL) and the others (SCR and SCY). In 20 yolk amino acid traits, there were moderate to high positive correlation coefficients each other, except for Y_Cys, Y_Asp, and Y_GABA. Among 18 albumen amino acid traits, moderate to strong positive phenotypic correlations were estimated, although there were no correlations between two traits (A_Gln and A_His) and the others.

In spite of positive correlations estimated among traits within each trait category (egg traits, yolk amino acid traits, and albumen amino acid traits), there were no or weak phenotypic correlations among the trait categories (Fig. 1). Interestingly, there were nearly no correlations between yolk and albumen amino acid traits even in the same compounds, which implying the different accumulating systems of amino acids into yolk and albumen. Moreover, moderate negative correlations were found between sizes and weights traits (EW, LLE, LSE, AW, and SW) and almost all albumen amino acid traits (e.g., A_Gly, A_Thr, A_Val, and A_Phe).

### Heterosis effect

**Figure 2:**
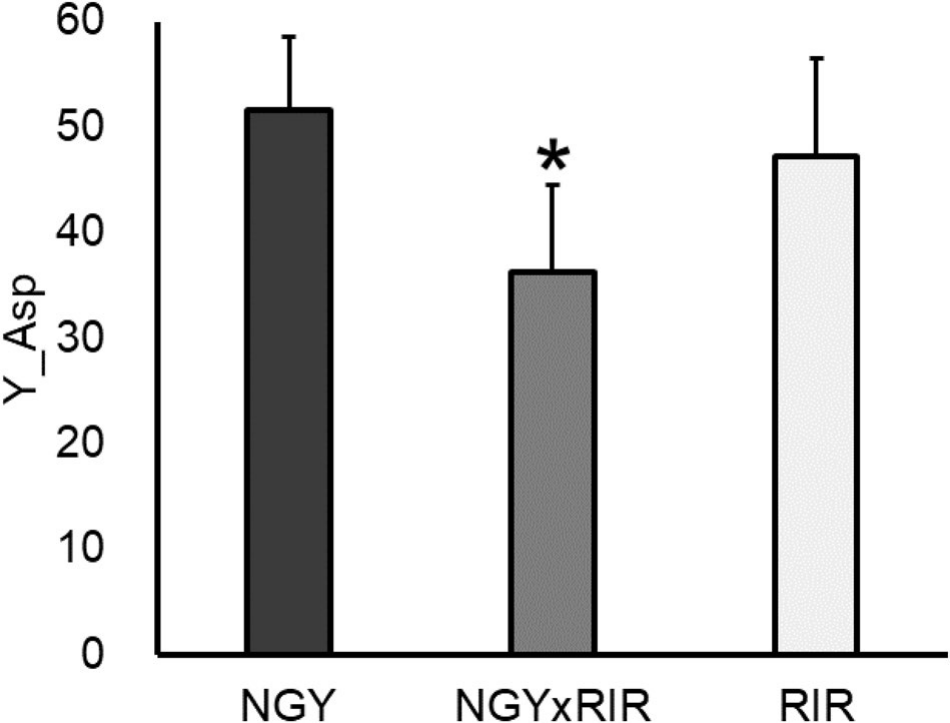
Heterosis effect on yolk aspartic acid trait. NGY, RIR, and their F_1_ hybrid (NGYxRIR) were used. Bar indicates standard deviation. One-way ANOVA and Tukey’s HSD test reveals significant effect on negative heterosis (*p* < 0.05) as indicated by an asterisk above F_1_ hybrid value.

To test heterosis effect on 48 traits, we used two sets of two parental breeds and their F_1_ hybrid (NGY, RIR, and NGYxRIR; SHA, RIR, and SHAxRIR). Tukey’s HSD tests revealed significant differences among NGY, RIR, and NGYxRIR on 23 traits (Supplementary Fig. 4). Especially, a significant effect on Y_Asp was found by one-way ANOVA (F_2,29_ = 9.77, *p* = 5.71E−04), and moreover Tukey’s HSD test discovered significant differences between parental breeds and their F_1_ hybrid (NGY vs NGYxRIR, *p* = 3.80E−04 and RIR vs NGYxRIR, *p* = 0.010), which indicating a significant negative heterosis (Fig. 2). On the other hand, using dataset of SHA, RIR, and SHAxRIR, significant differences in 24 traits were identified by Tukey’s HSD tests (Supplementary Fig. 5), but there were no heterosis effects.

## Discussion

This study aimed to reveal impact on genetic differences among various chicken breeds in 10 egg traits, 20 yolk amino acid traits, and 18 albumen amino acid traits using 5 breeds and 2 F_1_ hybrids. Of them, there were significant breed effects on 10 egg traits, 20 yolk amino acid traits, and 15 albumen amino acid traits. Moreover, a significant heterosis effect on Y_Asp has been identified in the comparisons among NGY, RIR, and NGYxRIR. In addition, positive correlations were estimated among traits within each trait category (egg traits, yolk amino acid traits, and albumen amino acid traits), whereas there were basically no or weak phenotypic correlations among the trait categories. However, moderate negative correlations were found between size and weight traits (EW, LLE, LSE, AW, and SW) and almost all albumen amino acid traits, but not in yolk amino acids. Thus, these results suggest that almost all egg traits and yolk and albumen free amino acid traits can be dramatically modified by genetic factor.

In general egg traits, five breeds including AUS, NGY, RIR, SHA, and UKO were investigated. Body weights of hens are 1.6 kg in AUS and 0.9 kg in UKO, whereas those of NGY, SHA, and RIR are around 3.0–4.5 kg^16,21^. Our previous report^16^ indicated that AUS produce comparable egg weight with RIR, although body weight in AUS is significantly lower than RIR. This study revealed that small size and weight of the egg from UKO will be corresponding to the small body size. This study also indicated that eggshell color of AUS, NGY, SHA, and UKO are tinted, whereas RIR produce brown eggshell color. In addition, F_1_ hybrids with the genetic background derived from RIR (NGYxRIR and SHAxRIR) produced brown eggs than the other breeds. Since these general egg traits including size, weight, and color of the egg have been mapped on several chromosomes^10–12^, further mapping studies with various breeds will identify more candidate genes for the regulation of the egg.

Yolk and albumen free amino acids were quantified to see effects on the genetic differences. In this study, completely the same mixed feed was fed to all hens from five breeds and two F_1_ hybrids. Rearing environment was also fixed in the conventional individual cages, and age at sampling of eggs was fixed as 62 wk of age. Therefore, major parts of the variation in the present results must be caused by genetic factors. In our previous study^16^, yolk cysteine was only significantly changed by breed difference using AUS and RIR. On the other hand, in this study, all yolk amino acids and almost all albumen amino acids were significantly differed by genetic differences. The ranking of almost all amino acid contents was seen as AUS > UKO, SHAxRIR, SHA > RIR > NGY, NGYxRIR in yolk and UKO, SHA > SHAxRIR, NGY > NGYxRIR > RIR, AUS in albumen. In both yolk and albumen amino acid traits, there are similar tendencies in the order, which indicating that UKO, SHA, and SHAxRIR showed higher contents, whereas RIR, NGY, and NGYxRIR indicated lower contents. However, a unique feature was found that AUS produced higher free amino acid contents in yolk but lower in albumen.

Essential amino acids for humans are known as 10 amino acids (Arg, His, Ile, Leu, Lys, Met, Phe, Thr, Tyr, and Val), while essential amino acids for poultry are these 10 amino acids plus two (Gly and Pro)^27^. In this study, several genetic background (UKO, SHA, and SHAxRIR) can be increased essential amino acid contents in both yolk and albumen, which suggesting that essential amino acids enriched eggs using genetics can be utilized for some specific needs e.g., alleviating malnutrition and supplying children and aged people. In addition, free amino acids are well known to contribute food taste^25^. Glu, Asp, Ala, and Tyr contribute umami taste, while Met, Ala, Gly, Pro, Ser, Val, and Lys affect sweet taste^26,28^. Although Asp, Glu, and Lys show sour taste, Pro, Gly, Val, Leu, Tyr, Phe, His, Lys, Ile, Arg, and Trp indicate bitter taste^26,28^. Asp and Lys affect salty and astringent taste, respectively^26,28^. Since these 16 taste-active amino acids can be altered by breeds, taste of yolk and albumen might be different. Given that egg taste can be changed by altered contents of free amino acids, taste added designer eggs will be provided in the egg industry. Moreover, egg components act as nutraceuticals, such like antioxidant properties, antimicrobial activities, immunomodulatory, anticancer, and antihypertensive activities^2,3^. This study revealed the impact on breed in yolk and albumen amino acids contents, and the previous studies have been reported that genetic factor can modify egg contents in fatty acids and minerals^13–15^. Therefore, it will be interesting to investigate the genetic factor in the bioactive fractions of eggs in future.

Phenotypic correlations among yolk and albumen free amino acids were estimated in this study. Although there were moderate or high positive correlations among the most amino acids traits in each yolk and albumen, no or weak correlations of amino acid contents were seen between yolk and albumen. These results suggested that there will be partially independent accumulating mechanisms of free amino acids into yolk and albumen. In addition, negative correlations were found among size and mass traits and albumen amino acid traits. This may imply that relatively low concentrations of albumen amino acids are found in larger eggs, since larger eggs tend to have higher volume of albumen.

This study revealed a heterosis effect on yolk aspartic acid (Y_Asp), indicating that F_1_ hybrid was significantly lower content than those of parental breeds (negative heterosis). In recent poultry industry, all commercial stocks utilize heterosis by crossing different strains^18^ to enhance performance in reproduction, growth, meat, and egg production using the benefits of a heterosis effect^23^. Comings and MacMurray^29^ summarized that hybrids combine the best traits of both parental strains and thus are more resistant to disease and other factors in several organisms. This study found the negative heterosis in yolk aspartic acid in a hybrid between NGY and RIR, but not in a hybrid between SHA and RIR. In Japan, there are many Jidori brands^23^ using hybrids between Japanese indigenous breeds and American breeds^21^. Therefore, several combinations of parental breeds should be tested in the future to find better combinations enhancing egg quality at molecular level.

This study clearly indicated that significant impacts on genetic differences among various breeds in yolk and albumen free amino acid contents. Many traits including morphology, behavior, and stress resistance traits have been studies well using animal models. Genetic architecture of quantitative traits is likely to be common feature in other species^30,31^. Amino acid contents in food stuffs have been investigated well in plant. Moderate to high heritability estimates and several QTLs for amino acid contents using genome-wide association study (GWAS) has been reported in Arabidopsis, soybean, and wheat^32–37^. These studies always suggested that complex genetic architecture of amino acid contents as well as the animal model^30,31^. Since there will be similar feature in genetic architecture of egg contents in chickens, further genetic analyses with large sample size will be needed.

## Methods

### Animals

Five breeds and two F_1_ hybrids of chickens in the Animal Research Center, Agricultural Research Department, Hokkaido Research Organization, Japan were used. Hokkaido Research Organization has kept all five breeds from generation to generation with more than 100 birds in each breed per generation by artificial insemination. Using the parental information, we carefully selected a wide variety of hens as possible in each group. A total of 81 hens from Australorp (AUS; n = 12), Nagoya (NGY; n = 12), Rhode Island Red (RIR; n = 12), Shamo (SHA; n = 11), Ukokkei (UKO; n = 12), F_1_ from a cross between NGY male × RIR female (NGYxRIR; n = 10), and F_1_ from a cross between SHA male and RIR female (SHAxRIR; n = 12) were investigated. Hens selected in each group will be representatives to see effect on genetic differences in egg traits. The hens were reared under the 16 h light and 8 h dark photoperiod cycle with free access to feeds and water in the individual cages^23^. Mixed feed for layers (Rankeeper; Marubeni Nisshin Feed Co., Ltd., Japan) was provided from 20 week of age. Detailed ingredients of the mixed feed were shown in Mori et al^16^. Daily management was conducted according to the rules of Standards Related to the Care and Management of Experimental Animals (Prime Ministers’ Office, Tokyo, Japan, 1980). This study was approved as authorization number 18–15 from the Experimental Animal Committee of the Obihiro University of Agriculture and Veterinary Medicine. All experiments were performed in accordance with relevant guidelines and regulations.

### Egg sampling and egg trait measurement

An egg from each hen (total of 81 eggs) was collected at 62 week of age. A total of 10 egg traits was measured. Egg traits included egg weight (EW), length of the long axis of egg (LLE), length of the short axis of egg (LSE), eggshell weight (SW), yolk weight (YW), albumen weight (AW), eggshell thickness (ST), eggshell color lightness (SCL), eggshell color redness (SCR), and eggshell color yellowness (SCY). Since all the egg traits measured are quantitative traits, it ideally requires much more samples. Weights were measured with an electronic balance (EK-6000H; A&D Company, Ltd., Japan). Two samples from each SHA and RIR lacked data of YW and AW, because yolk membrane was broken when yolk and albumen were divided. Sizes and eggshell thickness were measured using a digital caliper (P01 110-120; ASONE, Japan) and a Peacock dial pipe gauge P-1 (Ozaki MFG Co., Ltd., Japan), respectively. Eggshell colors were measured by a chromameter (CR-10 Plus Color Reader; Konica Minolta Japan, Inc., Japan). After measuring yolk weight, the yolk was diluted two-fold with distilled water (DW) and was mixed. The yolk solution and albumen were separately kept in tubes at − 30 °C until use.

### Yolk free amino acid analysis

Amino acid analysis for yolk was performed following the previous method^16^. Five milliliter of the yolk solution was mixed with 5 mL of 16% trichloroacetic acid solution (FUJIFILM Wako Chemicals, Japan) and vortexed. The samples were centrifuged at 1400×*g* for 15 min using a table-top centrifuge (model 2410; KUBOTA Corporation Co., Ltd., Japan) and a centrifuge (RX II series; HITACHI Ltd., Japan). After collecting the supernatant using a 1 mL syringe (NIPRO Corporation, Japan), the solution was filtered out to a microtube through a disposable cellulose acetate membrane filter unit with a 0.45 μm pore size (DISMIC-25CS; Advantec Toyo Kaisha, Ltd., Japan). The filtered solution was heated at 40 °C for 60 min in a vacuum oven (VOS-201SD, Eyela, Japan) until dry. After adding 20 μL of mixing solution (ethanol: DW: triethylamine = 2:2:1), the microtube was vortexed for 20 min using a Micro Mixer E-36 (TAITEC Corporation, Japan). The sample was heated at 40 °C for 60 min in a vacuum oven. After drying, 20 μL of mixing solution (Ethanol: DW: triethylamine: phenylisothiocyanate = 7:1:1:1) was added and mixing for 20 min using a micro mixer. The sample was re-heated at 40 °C for 60 min in a vacuum oven for drying. After preprocessing, the sample tube was placed at − 30 °C until the sample was analyzed.

Yolk amino acids were analyzed by HPLC (LC-2010CHT; Shimadzu Co. Ltd., Japan). Following the same method for sample preprocessing, amino acid standards (types H and B), l-asparagine, and l-glutamine (FUJIFILM Wako Chemicals, Japan) were prepared. The standards were analyzed before every 30 samples. The absolute concentration of amino acids in yolk was calculated from the peaks of sample and standard.

### Albumen free amino acid analysis

Albumen (100 mg) was vortexed for 1 min with 800 µL of 0.1 M HCl. Hexan (800 µL) was added and vortex mixed for 1 min. After centrifuging at 22,000×*g* for 5 min, the lower layer was collected. The lower layer (400 µL) was mixed with 1200 µL of water‐acetonitrile (1:2 v/v) by vortexing for 1 min. After centrifuging at 22,000×*g* for 5 min, the supernatant was filtered through a 0.2 µm pore size filter.

Albumen amino acid was analyzed by the UHPLC system (NexeraX2, Shimadzu Co., Ltd., Japan) using a Kinetex 2.6 µm column (EVO C18; 100 × 3.0 mm). Gradient elution program was applied using two mobile phases, which are mobile phase A (17 mM of potassium dihydrogenphosphate and 3 mM of dipotassium hydrogen phosphate) and mobile phase B (mixing solution of DW: acetonitrile: methanol = 15:45:40). Precolumn derivatization was performed by the UHPLC system with 45 µL of mercaptopropionic acid, 22 µL of orthophthalaldehyde, and 7.5 µL of samples. After placing the mixture for 2 min, 5 µL of fluorenylmethyl chloroformate was mixed and placed for 2 min. Then, the sample (1 µL) was injected into the column. Flow rate was set 0.85 mL/min and column temperature was set 35 °C. An RF-20Axs high-sensitivity fluorescence detector was used at Ch1 (Ex 350 nm and Em 450 nm) and Ch2 (Ex 266 nm and Em 305 nm).

Standard solution of amino acids mixture type H, l-asparagin, l-glutamine, l-cystine, and l-tryptophan (FUJIFILM Wako Chemicals, Japan) were preprocessed following the same method for albumen samples. Calibration curve was calculated by serial dilution of the standards. The absolute concentration of albumen amino acids was calculated from the curve.

### Statistical methods

In order to test the effect on genetic differences in egg traits and yolk and albumen amino acid traits, data were analyzed using one-way analysis of variance (ANOVA) with significant threshold (*p* < 0.05). For testing heterosis effect, two sets of two parental breeds and their F_1_ hybrids (NGY, RIR, and NGYxRIR; SHA, RIR, and SHAxRIR) were used. Tukey’s honestly significant difference (HSD) test was conducted to see inter-breed differences (*p* < 0.05). Data are indicated as the mean and standard deviation. Statistical analyses were performed using R software^38^. Pearson’s correlations among all traits were calculated and plotted using ‘corrplot’ package of R. Significant threshold (*p* < 0.05) was used to see whether or not correlation coefficients equal zero.

## Supplementary Information

Supplementary Information.

## Data Availability

The datasets generated during the current study are available from the corresponding author on reasonable request.
