# Supplementary material for: Impact on genetic differences among various chicken breeds on free amino acid contents of egg yolk and albumen
Source: Sci Rep. 2021 Jan 26;11:2270. doi: 10.1038/s41598-021-81660-3 (PMC7838262; doi:10.1038/s41598-021-81660-3)
Supplement: Supplementary file 1 — Supplementary Information. [file 41598_2021_81660_MOESM1_ESM.docx]

**Impact on genetic differences among various chicken breeds on free amino acid contents of egg yolk and albumen**

Tatsuhiko Goto^1,2*^, Saki Shimamoto^3,4^, Masahiro Takaya^2,5^, Shun Sato^6^, Kanna Takahashi^2^, Kenji Nishimura^2^, Yasuko Morii^6^, Kyoko Kunishige^6^, Akira Ohtsuka^3^, Daichi Ijiri^3^

^1^ Research Center for Global Agromedicine, Obihiro University of Agriculture and Veterinary Medicine, Obihiro, Hokkaido 080-8555, Japan

^2^ Department of Life and Food Sciences, Obihiro University of Agriculture and Veterinary Medicine, Obihiro, Hokkaido 080-8555, Japan

^3^ Department of Biochemical Science and Technology, Kagoshima University, Korimoto, Kagoshima 890-0065, Japan

^4^ Graduate School of Science and Technology, Niigata University, Niigata 950-2181, Japan

^5^ Hokkaido Tokachi Area Regional Food Processing Technology Center, Tokachi Foundation, Obihiro, Hokkaido 080-2462, Japan

^6^ Agricultural Research Department, Animal Research Center, Hokkaido Research Organization, Shintoku, Hokkaido 081-0038, Japan

**Corresponding author:** Tatsuhiko GOTO, Ph.D.; Research Center for Global Agromedicine, Obihiro University of Agriculture and Veterinary Medicine, Obihiro, Hokkaido 080-8555, Japan. [tats.goto@obihiro.ac.jp](mailto:tats.goto@obihiro.ac.jp)

**Supplementary information**

**Figure S1. Results of Tukey’s HSD test in 10 egg traits using five breeds and two F_1_ hybrids.**

All combinations among breeds were tested. Trait abbreviations are expressed in Materials and Methods. P value is shown in each cell. Cells filled by orange meant significant difference in each pair (P < 0.05).

**Figure S2. Results of Tukey’s HSD test in 20 yolk egg amino acid traits using five breeds and two F_1_ hybrids.**

All combinations among breeds were tested. Trait abbreviations are expressed in Materials and Methods. P value is shown in each cell. Cells filled by orange meant significant difference in each pair (P < 0.05).

**Figure S3. Results of Tukey’s HSD test in 18 albumen egg amino acid traits using five breeds and two F_1_ hybrids.**

All combinations among breeds were tested. Trait abbreviations are expressed in Materials and Methods. P value is shown in each cell. Cells filled by orange meant significant difference in each pair (P < 0.05).

**Figure S4. Results of Tukey’s HSD test in all 48 traits using NGY, RIR, and their F_1_ hybrid.**

All combinations among breeds were tested by Tukey’s HSD tests. Trait abbreviations are expressed in Materials and Methods. P value is shown in each cell. Cells filled by orange meant significant difference in each pair (P < 0.05). Red letters indicate a heterosis effect.

**Figure S5. Results of Tukey’s HSD test in all 48 traits using SHA, RIR, and their F_1_ hybrid.**

All combinations among breeds were tested by Tukey’s HSD tests. Trait abbreviations are expressed in Materials and Methods. P value is shown in each cell. Cells filled by orange meant significant difference in each pair (P < 0.05).

**Figure S1**

**Figure S2**

**Figure S3**

**Figure S4**

**Figure S5**
